# Contact Nonlinear Acoustic Diode

**DOI:** 10.1038/s41598-020-59270-2

**Published:** 2020-02-13

**Authors:** Yao Huang, Xiaoyu Wang, Xun Gong, Haodong Wu, Dong Zhang, De Zhang

**Affiliations:** Key Laboratory of Modern Acoustics, MOE, Institute of Acoustics, Department of Physics, Collaborative Innovation Center of Advanced Microstructures, Nanjing University, Nanjing, 210093 China

**Keywords:** Acoustics, Nonlinear phenomena

## Abstract

Nonlinear implementations of acoustic diodes are inherently nonreciprocal and have received continuous attention from the beginning of the research boom for acoustic diodes. However, all the reported nonlinear schemes usually have the shortcomings such as low transmission ratio, action threshold, lack of stability and cumbersome setups. In the present design, we take advantage of extraordinarily large contact acoustic nonlinearity which is several orders of magnitude stronger than material nonlinearity. It is theoretically found that the spectra of the transmitted wave depend on the contact time. It is proven experimentally that the contact nonlinearity can be tamed by adjusting the driving amplitude, the static stress and the elastic constants of the materials. In order to build a compact acoustic diode, a sub-wavelength filter with a sandwich structure is designed. The total length of the acoustic diode is only three eighths of the incident wavelength. The amplitude-dependent behavior of the device exhibits similarities with electronic diodes. A more than 50% transmission ratio is obtained. A robust, stable, compact, highly efficient and solid-state acoustic diode is realized.

## Introduction

Reciprocity is a fundamental property of the acoustic wave, which means the acoustic wave inherently travels symmetrically in space. Devices of one-way transport for acoustic wave which are called as acoustic diodes, isolators or rectifiers are of great importance in acoustic engineering^1^.

Recent years have witnessed a lot of researches addressing on design of a variety of one-way transport acoustic devices. Among them, a rather number of designs are making use of asymmetric scatters^2,3^, mode conversion in wave guides^4,5^, angular-dependent band gap in phononic crystal^6–13^, or abnormal reflection and refraction of metamaterials^14–18^. Strictly speaking, most of these designs don’t break the reciprocity when all modes and all ports are considered together. However, obviously these schemes may still be useful in some scenarios such as noise insulation etc. There are also some designs that take advantage of active elements^19,20^. Due to the powerful ability of the combination of actuators or transducers with the electronic circuit, these designs do break the reciprocity and have excellent performance of one-way transport, but the introduction of extra elements make them so different to their passive counterparts and limit the range of their applications.

Theoretically, there are two classes of non-conventional topologies that can be adopted to break the reciprocity and realize one-way transport of acoustic waves. 1) The first class of topologies applies extrinsic stimuli to modulate the properties of the host medium in space, time or both of them^21–24^. For examples, R. Fleury *et al*. introduced uni-rotational fluid circulations into an acoustic circulator^21^. H. Nassar *et al*. modulated the elastic moduli and mass density in time and space in a wavelike fashion^24^. These approaches have the advantage of invariant frequency. However, external stimuli may introduce noise and absorption losses. Like the active designs, auxiliary devices and consumption of extra energy are needed. 2) The second class of topologies involves taking advantage of intrinsic properties of the media such as structural chirality^25–27^, dissipation^17,28^ and nonlinearity^29–35^. For example, C. He *et al*. elegantly realized the inversion of acoustic energy bands at a double Dirac cone, and experimentally demonstrated an acoustic topological insulator and robust one-way sound transport^24^. Due to the two-dimensional acoustic beam of the edge mode, such isolators may have difficulty in manipulating three-dimensional acoustic beams. On the other hand, it is very difficult to excite the edge mode efficiently. Of course, these devices may be suitable for on-chip phononic circuits.

A nonlinear system is inherently nonreciprocal. Several nonlinear schemes have been suggested and implemented^29–35^. For example, Liang *et al*. realized one-way acoustic propagation by using a superlattice as a filter coupled with bubble suspension water as a frequency converter^29,30^. N. Boechler *et al*. exploited bifurcations and chaos in a granular crystal for tunable rectification^31^. Thibaut Devaux *et al*. investigated an acoustic rectifier composed of a phononic crystal as a filter and a nonlinear granular material as a harmonic generator^32^. F. Li *et al*. realized granular acoustic switches and logic elements^33^. However, all the reported nonlinear systems usually have the shortcomings such as low transmission ratio (the ratio between the transmitted wave amplitude to that of the incident wave), action threshold, lack of stability and cumbersome setups. C. Liu *et al*. presented a theoretical proposal of nonlinear phononic crystal so as to build a frequency-preserved acoustic diode with high forward-power-transmission rate (the ratio of the acoustic flux of the transmitted wave to that of the incident wave)^34^. However, it is difficult to be materialized.

For a nonlinear acoustic diode, it is usually composed of two parts: a part of nonlinear material and a super lattice. The nonlinear material functions as a frequency converter which can generate super-harmonics, sub-harmonics, difference frequency waves or sum frequency waves. The super lattice acts as a filter which blocks the fundamental wave but lets the frequency-shifted wave pass. When the acoustic wave comes from the side of the super lattice, it will be blocked, and no wave can pass the device. When it impinges on the nonlinear material first, frequency-shifted waves occur and pass through the super lattice. The transmission ratio is mainly determined by the conversion efficiency of the nonlinear part. Congyi Fu *et al*. used two masses connected by a bilinear spring with asymmetric tension and compression stiffness as the converter^36^. However, their design is suitable for insulation of vibration, but not for control of wide acoustic beam. It is well known that anomalously high nonlinearity has been found in solids with defects, such as cracks, grains or delaminations, which are referred to as contact acoustic nonlinearity (CAN). The effective nonlinear coefficients can be very high, and they are several orders of magnitude larger than material nonlinearity. Strong second harmonics can be observed. The nonlinear schemes made of granular material essentially take advantage of the CAN effect between grains. Itay Grinberg *et al*. proposed two nonlinear schemes with vibro-impact elements theoretically^37^. In fact, a solid-solid contact interface is the simplest vibro-impact element. However, the contact acoustic nonlinear phenomena are considered to be elusive, uncontrollable, and unpredictable. For CAN, the elastic properties of materials on both sides of the interface, the evenness of the contact surfaces, static stress and the driving level are all influential factors. If the contact acoustic phenomena are controllable, the contact interface itself can acts as the frequency converter. Thus, the space occupied by the nonlinear material in the aforementioned nonlinear schemes is no longer needed, and a compact, stable, highly efficient, solid-state acoustic diode becomes possible. Because of the low attenuation factor in solids, high transmission ratio is also expected.

**Figure 1:**
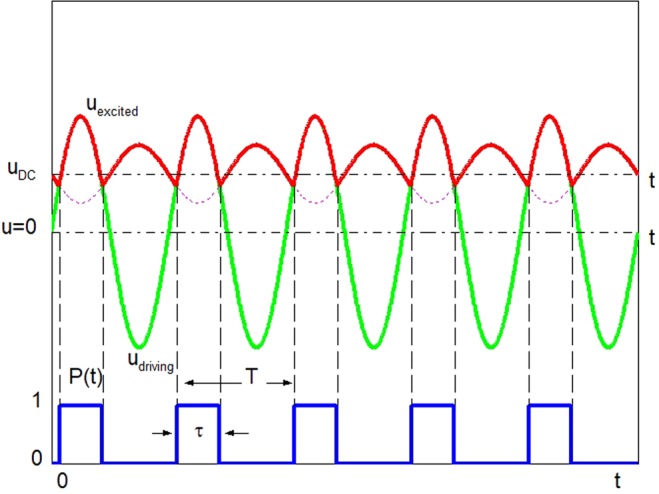
CAN-phenomenological model: the driving vibration and the excited vibration at the interface. The blue line is the square wave *p(t)*. The green line is the displacement of the driving surface *u*_*driving*_. The red line is the displacement of the driven surface *u*_*excited*_.

What determines the spectra of the transmitted wave? How to get the strongest second harmonic? Referring to the work of I. Y. Solodov *et al*.^38^, the effect of the contact time on the spectra of the transmitted wave is analyzed. In their CAN-phenomenological model, strain is used to describe the driving mechanism at the interface. In fact, it is the ‘displacement’, not the ‘strain’ of one side that drives the other side to move, so the displacement is used in the following analysis. The displacement of the driving surface is *u*_*driving*_ = *A*_*driving*_*cos(ω*_*0*_*t)*. During each cycle, the two sides will contact for a period of time *τ*. The contact time depends on the vibration amplitude of the driving surface and the DC component of the vibration *u*_*DC*_ of the driven surface. This modified CAN-phenomenological model is shown in Fig. 1. *T* is the period of the vibration of the driving surface. The impact the driven side received can be expressed as the sinusoidal wave *u*_*driving*_ multiplied by a square wave *p(t)*. The spectrum of the transmitted wave is a convolution of the spectra of *u*_*driving*_ and *p(t)*. The corresponding frequency spectra of *u*_*driving*_ and *p(t)* are *F*_1_ and *F*_2_ respectively.

1 $${U}_{excited}(m{\omega }_{0})={F}_{1}[{u}_{{\rm{driving}}}(t)]\ast {F}_{2}[p(t)]$$

*F*_1_ and *F*_2_ are well known,

2a $${F}_{1}=F[{\cos }({\omega }_{0}t)]=2\pi (1/2\delta (\omega +{\omega }_{0})+1/2\delta (\omega -{\omega }_{0})).$$

2b $${F}_{2}=F(p(t))=p(n{\omega }_{0})=2\pi \tau /T{\sum }_{n=-\infty }^{\infty }\exp (-jn{\omega }_{0}T/4)Sinc(n{\omega }_{0}\tau /2)\delta (\omega -n{\omega }_{0}).$$

**Figure 2:**
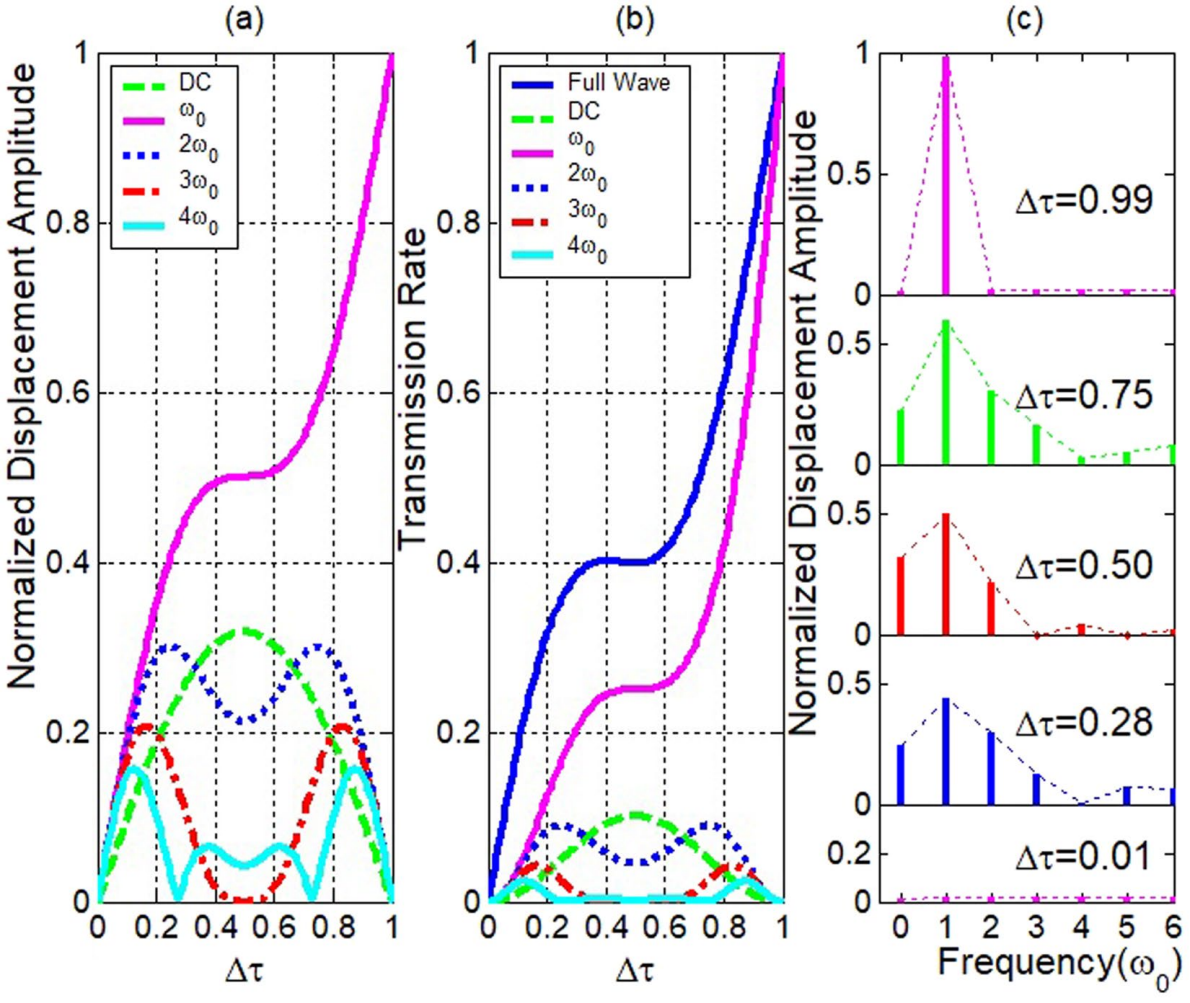
Relation of the spectra of the transmitted waves with the contact time. (**a**) The normalized displacement amplitudes of each frequency component of the transmitted wave vs. the contact time. (**b**) The transmission rate of each frequency component of the transmitted wave vs. the contact time. (**c**) The spectra of the transmitted wave for several contact times *Δτ* = 0.01, 0.28, 0.5, 0.75 and 0.99.

By combining them in convolution sum,

$${U}_{excited}(m{\omega }_{0})=\pi \tau /T{\sum }_{n=-\infty }^{\infty }Sinc(n\pi \tau /T)exp(-j(n-1)\pi /2)$$

3 $$(\delta (m{\omega }_{0}-(n-1){\omega }_{0})-\delta (m{\omega }_{0}-(n+1){\omega }_{0})).$$

where *m* and *n* are integer numbers from −∞ to ∞. δ*(mω*_*0*_ − *nω*_*0*_) is the unit pulse function. *ω*_*0*_ is the round frequency of the vibration of the driving surface. The spectra of the transmitted waves are calculated with the above equation and shown in Fig. 2, where *Δτ* = *τ/T*.

The spectra of the nonlinear vibrations (Eq. 3) are shown in Fig. 2. It demonstrates some unusual CAN features. It contains several super harmonics. In Fig. 2(a), *Δτ* = 0.5 is the symmetry center. The fundamental component is anti-symmetric. Other components including the DC and super harmonics are symmetric. For a *Δτ* and its counterpart 1 − *Δτ*, the spectra contain similar components of super harmonics. With the increasing of *Δτ*, the fundamental wave increases monotonically, but the DC component and the super harmonics have peaks and dips. The higher the order is, the more the peaks and dips there are. Between *Δτ* = 0.11 and *Δτ* = 0.89, as for the amplitude, the second harmonic is no less than 20% of the incident fundamental wave. At *Δτ* = 0.25 and *Δτ* = 0.75, the second harmonic is about 30% of the incident fundamental wave. In fact, the DC component is the average vibration equilibrium displacement. However, at the peak of the DC component around *Δτ* = 0.5, because there is a rather long period of time when the super harmonics and the fundamental wave are out of phase, there is a dip for the super harmonics. In Fig. 2(b), the transmission rate (the square value of the normalized amplitude of the fundamental or each of its harmonics) is shown. The amplitudes of harmonics are always modulated by the Sinc function envelope as shown in Fig. 2(c). This seriously affects the dynamic behavior of the CAN spectrum. As *Δτ* decreases from 1 to 0, it is accompanied by the correspondent “compression” of the envelope function. The above spectral features are summarized in an unusual (rectified sine) waveform distortion due to the CAN. The contact time is determined by the driving amplitude, the initial stress and the elastic constants of the materials on both sides of the interface. So, through adjusting these three parameters, a strong second harmonic is obtainable.

The constants of the elasticity of both sides influence the contact time very much. Large nonlinearity can be readily observed on an interface between a hard and a soft material. However, for a soft material such as silicone rubber, given an initial static stress, there will be a large initial static strain. Therefore, it’s hard for the two sides to separate. That’s why rubber is often used as a sealing material. In fact, if there is no static stress, a long separation time is possible and large CAN occurs. However, the mismatch of impedance between a hard and a soft material leads to large reflection and a little transmission at the interface. Moreover, adhesion between a soft and hard material is stronger than that between two hard materials, which means it is more difficult for the two sides to separate. So, an interface between a hard and a soft material is not chosen to build the acoustic diode. It’s also imaginable that if both sides are of the same material, it is difficult for them to separate. So, as a compromise of conversion efficiency, transmission ratio and stability, two different but similar materials are desirable. In the experiments, 6061 and 5052 duralumin are chosen to build the diode. The longitudinal wave velocities of them are 6413 m/s and 5882 m/s respectively. The densities of them are 2732 kg/m3 and 2596 kg/m^3^ respectively.

**Figure 3:**
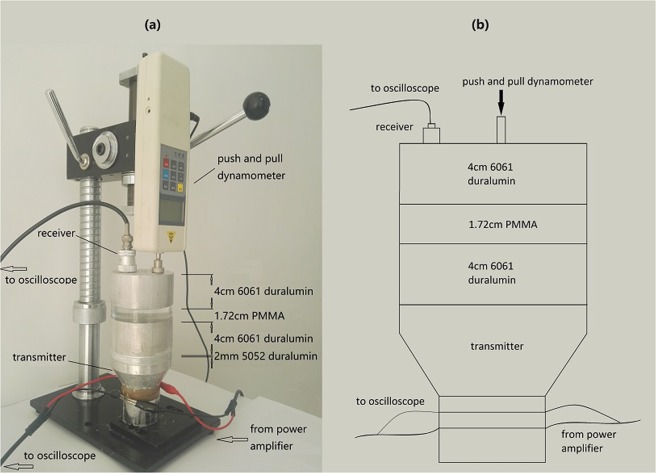
(**a**) The photograph of the experimental setup for contact nonlinear acoustic diode. (**b**) Schematic of the experimental setup for contact nonlinear acoustic diode.

**Figure 4:**
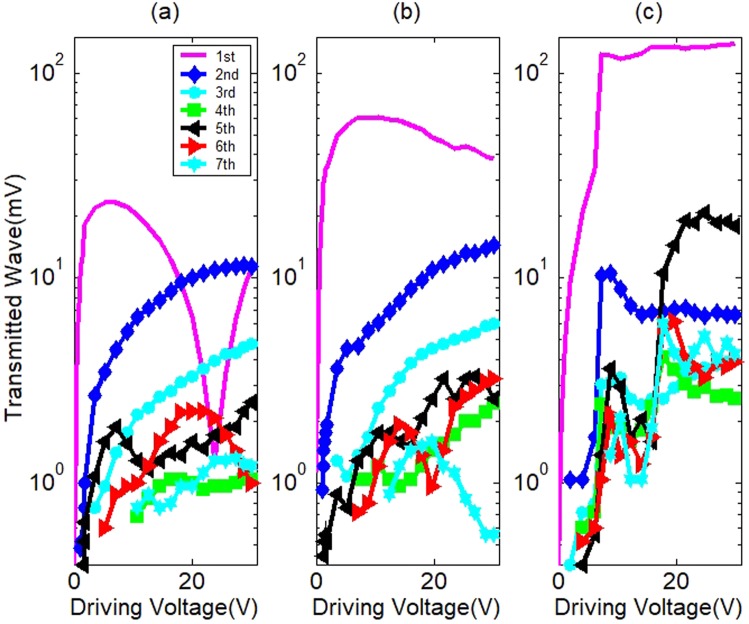
Spectra of the transmitted wave with the driving voltage under external force (**a**) 0 N, (**b**) 4.1 N and (**c**) 9 N.

The initial static compression stress *T*_*0*_ which leads to initial strains on both sides of the interface keeps the two surfaces in touch for a longer time in each cycle. In the following, an experiment is carried out to investigate the influences of the initial static stress and the driving level on the spectra of the transmitted wave. The experimental setup is shown in Fig. 3. It consists of a transmitter, a round 2 mm thick 5052-duralumin sheet, a 2 cm long 6061-duralumin cylinder, a receiver and a push and pull dynamometer. The sandwich structure shown in Fig. 3 is for the following acoustic diode experiment. In this experiment, only a 2 cm long 6061-duralumin cylinder is used. To save space, only one figure is shown for the two experiments. The receiver is fixed on the top surface of the 6061-duralumin cylinder. It is a home-made piezoelectric composite transducer. The transducer operates as a capacitive transducer at frequency of tens of kilohertz which is far away from the center frequency 2 MHz. Though its response at tens of kilohertz is small but flat with the frequency. The transmitter is a piezoelectric transducer from Suzhou Hairui Electronics Technology Company. The surface of the transmitter is coarse, so the sheet is bonded perfectly to the transmitter to get a smooth surface. The sheet is in direct contact with the cylinder. The cylinder is bonded perfectly to the receiver. Sodium salicylate is used in the bonding process. The dynamometer presses on the top of the cylinder. The aluminum sheet is very thin, so its influence on wave transmission can be ignored. The spectrum distribution of the transmitted wave is measured with external force of 0 N, 4.1 N and 9 N. The frequency of the incident wave is 20 KHz. Of course, the aluminum cylinder’s weight of 2.63 N also plays a role. The driving level increases from 0 to 30 V. The results are shown in Fig. 4.

The external force makes the interface more tightly coupled, with more acoustic flux passing through the interface. In this respect, it is similar to adhesion. The fundamental wave under 9 N is the strongest. The fundamental wave under no external force is the weakest. The harmonics in fact depend on both the external force and the driving level. For a weak driving level, the interface cannot be separated and there are no super harmonics. For a strong driving level, both the acoustic flux passing through the interface and the reflected acoustic flux become strong and the interface begins to clap and kiss, and super harmonics occur. If the compression stress is big enough, the two surfaces will not separate, and no harmonic is generated. The transmitted wave will be the fundamental wave only. After the driving voltage surpasses a certain threshold, super harmonics appear. In Fig. 4, the threshold values are 0.51 V, 1.01 V and 2.02 for 0 N, 4.1 N and 9 N respectively. With the driving level’s increasing, Δ*τ* decreases from 1 and the separation time increases. However, in Fig. 4(a) for 0 N, the transmitted fundamental wave has a dip around 23.9 V which is different to the results in Fig. 2(a,b). Following is our explanation about the phenomena. Driven by the transducer, the duralumin cylinder will bounce up, and fall down freely due to the gravity. The time of the up and down movement depends on the initial upward velocity of the duralumin resulted from the impact of the transducer. The strength of the impact is proportional to the driving voltage. When the driving voltage increases to a certain value, the duralumin cylinder will move up and down steadily for the same period as the incident acoustic wave. At the same time, the transmitted fundamental wave decreases to its minimum. With the driving voltage increasing further, the period of the up and down movement become longer than that of the incident wave. Thus, the amplitude of the up and down movement decreases, and the transmitted fundamental component increases again. In Fig. 4(a), an anti-resonant dip around 23.9 V can be found for the fundamental component. The amplitudes of the high order harmonics depend on the contact time and the driving voltage, and they are not subject to the same dissipation as the fundamental component. However, this phenomenon is sensitive to external conditions. When there is an initial static compression stress, the duralumin cylinder cannot move up and down freely and such dip can hardly be observed. In most cases, results like Fig. 4(b) for 4.1 N are observed. If the driving amplitude continues to increase to a rather large value, the transmitted wave reaches to a saturation state where its amplitude increases very slowly or even decreases a little. This can be interpreted as that the stronger the driving level, the bigger the DC component of the transmitted wave which means a shorter contact time and a weaker transmitted acoustic flux. With the driving level increasing further, the sub-harmonics will appear. In the end, fraction and chaos will occur. In the experiment, the nonlinearity of the transducer and signal-amplifier will become obvious when the driving voltage reaches 30 V. The initial static compression stress can contribute to the stability of the system and delay the occurrence of sub-harmonics and chaos. The experiment proves that a strong output of the second harmonic can be obtained by adjusting the initial compression stress and the driving amplitude. Here, it should be noted that the system is nonlinear. Therefore, the relation between stress and strain is a complex hysteresis curve. Even in one cycle, the historical state of stress action is different at each time, and it is impossible to expect a definite corresponding strain response for the stress. Especially, when the system works at such a high frequency, it is impossible to investigate the hysteresis effect of the system, and it is feasible and meaningful to investigate the overall input-output characteristics of the system.

**Figure 5:**
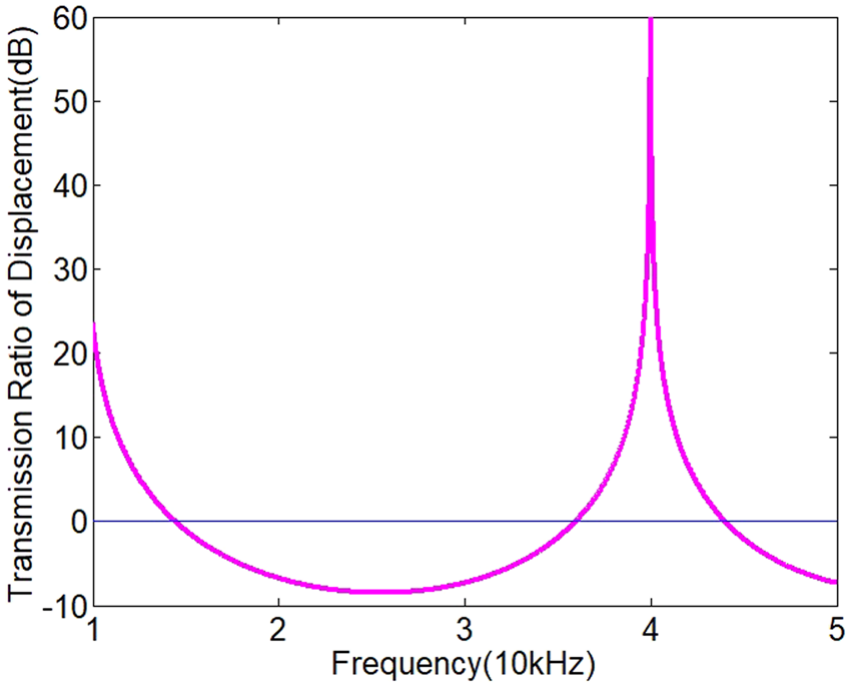
The transmission ratio of displacement with frequency.

In order to realize an acoustic diode, an efficient and compact filter is also needed. A sub-wavelength filter with a sandwich structure is designed. It is simply composed of three parts: a 6061-duralumin cylinder, a PMMA (polymethyl methacrylate) cylinder and another 6061-duralumin cylinder. The three parts are bonded together with epoxy resin. The thickness of each part is one eighth of the wavelength of the fundamental wave at 20 kHz. The total length of the filter is only 3 eighths of the wavelength of the incident wave. Due to using the contact nonlinear acoustic effect, no extra space for nonlinear material is needed and this size is just the total size of the acoustic diode. The longitudinal wave velocity and the density of the PMMA are 2763 m/s and 1187 kg/m^3^ respectively. The thicknesses of the PMMA and duralumin cylinder are 17.3 mm and 40 mm respectively. The total length of the acoustic diode is 97.3 mm. The relationship of transmission ratio of displacement with frequency is analyzed by transmission line theory^39^ and shown in Fig. 5.

There is a resonance at 40 KHz. The insertion loss at 20 KHz is 6.7 dB. So, it can depress the fundamental wave and let the second harmonic pass. The frequency range where the transmission ratio is over 0 dB is the pass band. The frequency range where the transmission ratio is below 0 dB is the stop band. It can be found that the pass bandwidth around 40 KHz is about 8 KHz and the stop bandwidth is about 20 KHz. So, this filter can be considered as a wide band filter. Though the following experiment on the acoustic diode is done at a single frequency because of the narrow band transmitter, the acoustic diode should have a rather wide working band. The center of the stop band is around 25 KHz.

**Figure 6:**
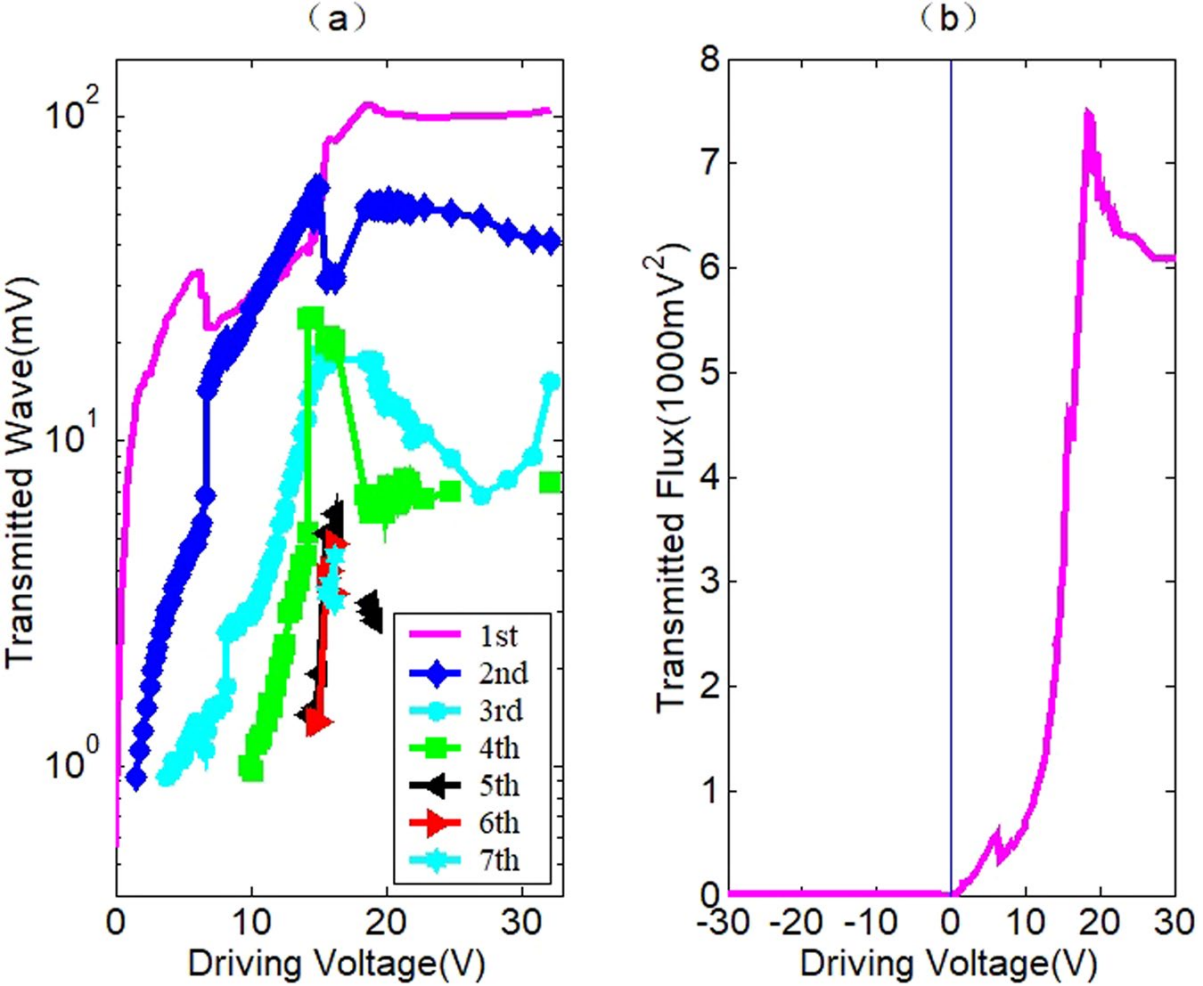
The transmission characteristics of a contact nonlinear acoustic diode. (**a**) Spectra of the transmitted wave with the driving voltage under zero external force. (**b**) The amplitude-dependent transmission behavior.

Following is about the experiment on the acoustic diode. The experiment setup can be referred to Fig. 3. There is no external force in the experiment. Figure 6 illustrates the experimental results of the acoustic diode. The amplitude-dependent behavior of the proposed device exhibits similarities with electronic diodes as shown in Fig. 6(b). The negative voltage is used to describe the backward transmission. It can be observed that the backward transmission is almost completely stopped. When the driving level is small, −2V to 0 V, no transmission can be observed. Even at a high driving level, such as −30V, the transmitted wave is only ignorable 2.6 mV which can still be reduced further with more precise manufacturing and optimization of the components of the filter. On the contrast, the forward transmission is very strong. The forward transmission is around 10000 times stronger than the backward transmission. In Fig. 6(b), the total transmitted acoustic flux (the sum of the square values of the amplitudes of the fundamental and its harmonics) is plotted against the driving voltage. Due to the perfect prohibition effect on the incident fundamental wave for backward transmission, not only the second harmonic but the full wave acoustic flux can be considered as effective forward transmission. The forward transmission increases with the driving voltage until about 20 V. Then, it becomes saturation. The self-weight of the filter which is about 11.5 N unavoidably exerts pressure on the contact interface. According to the results of Fig. 4, in the situation of so big an external force, for the forward transmission, a strong transmitted fundamental wave, weak super-harmonics and high CAN threshold are expected. However, due to the strong rejection effect of the filter which means high input impedance, or a high potential barrier for the incident fundamental wave, it strongly reflected at the contact interface, and strong contact acoustic nonlinearity occurs with a lower threshold value of 1.57 V as shown in Fig. 6(a). The harmonics are much stronger than those for a common contact interface in the former experiment. When the working threshold of the acoustic diode is considered, the value is zero as shown in Fig. 6(b). The second harmonic reaches its climax when the driving level is about 15 V. Corresponding to the climax, the contact time *Δτ* = 0.75 and the transmitted acoustic energy flux is about 54% of the incident wave can be gotten from Fig. 2(b,c). With the increasing of the contact time, the transmitted acoustic flux will increase too. Contrary to the backward transmission, the depression effect for the transmitted wave of rich harmonic components is greatly weakened. For the second harmonic, there is even amplification due to the resonance. Theoretically, for a continuous wave of the frequency at the rejection band center, the forward wave and the reflected wave in the filter are always out of phase and they cancel each other. So, the backward transmission is almost completely prohibited. However, in the case of forward transmission, since there is a separation time in each cycle of the incident wave in addition to the above-mentioned rich harmonics which means a complex waveform, the phase relation of the waveform and its reflection is almost random. So, the forward wave can hardly be reduced. The filter acts like a fish trap. The waves that enter the filter propagate forwards with little reduction. Due to the similarity of the duralumin 6061 and 5052, the contact surfaces keep in touch for a long time each cycle and the gravity force of the filter, adhesion or a proper external force will make the contact time even longer. As a result, most incident acoustic flux enters the filter and leads to a strong forward transmission. A more than 50%, perhaps up to 80% transmission ratio is realizable according to the simulation results in Fig. 2(b). It is impossible for the previous nonlinear acoustic diodes to obtain so large a transmission value. Moreover, if the driving level is rather high, the CAN will lead to fractions and chaos. Nevertheless, due to the efficient rejection effect of the filter for a backward transmission, even at the state of fractions and chaos, the device can still function as an acoustic diode. This device does relay on frequency conversion, and therefore, results in signal distortion when transmitting through it in the forward direction. Though this may limit its application to some specific areas, there is no doubt that a robust, stable, compact, highly efficient and solid-state acoustic diode has been realized.
